# Rosemary (*Rosmarinus officinalis*) extract causes ROS-induced necrotic cell death and inhibits tumor growth *in vivo*

**DOI:** 10.1038/s41598-018-37173-7

**Published:** 2019-01-28

**Authors:** Almudena Pérez-Sánchez, Enrique Barrajón-Catalán, Verónica Ruiz-Torres, Luz Agulló-Chazarra, María Herranz-López, Alberto Valdés, Alejandro Cifuentes, Vicente Micol

**Affiliations:** 1 Instituto de Biología Molecular y Celular (IBMC) and Instituto de Investigación, Desarrollo e Innovación en Biotecnología Sanitaria de Elche (IDiBE), Universitas Miguel Hernández (UMH), 03202 Elche, Spain; 2 Laboratory of Foodomics, Institute of Food Science Research (CIAL, CSIC), Nicolas Cabrera 9, 28049 Madrid, Spain; 3 CIBER, Fisiopatología de la Obesidad y la Nutrición, CIBERobn, Instituto de Salud Carlos III (CB12/03/30038), 07122 Palma Sola, Spain

## Abstract

Colorectal cancer is the third most common diagnosed cancer globally. Although substantial advances have been obtained both in treatment and survival rates, there is still a need for new therapeutical approaches. Natural compounds are a realistic source of new bioactive compounds with anticancer activity. Among them, rosemary polyphenols have shown a vast antiproliferative capacity against colon cancer cells *in vitro* and in animal models. We have investigated the antitumor activity of a rosemary extract (RE) obtained by using supercritical fluid extraction through its capacity to inhibit various signatures of cancer progression and metastasis such as proliferation, migration, invasion and clonogenic survival. RE strongly inhibited proliferation, migration and colony formation of colon cancer cells regardless their phenotype. Treatment with RE led to a sharp increase of intracellular ROS that resulted in necrosis cell death. Nrf2 gene silencing increased RE cytotoxic effects, thus suggesting that this pathway was involved in cell survival. These *in vitro* results were in line with a reduction of tumor growth by oral administration of RE in a xenograft model of colon cancer cells using athymic nude mice. These findings indicate that targeting colon cancer cells by increasing intracellular ROS and decreasing cell survival mechanisms may suppose a therapeutic option in colon cancer through the combination of rosemary compounds and chemotherapeutic drugs.

## Introduction

Colorectal cancer (CRC) is the second most commonly diagnosed cancer type in females and the third in males globally, with increasing prevalence even in traditionally low-risk countries. Nevertheless, a decrease in colorectal cancer mortality rates have been noticed in a large number of countries, most probably due to reduced prevalence of risk factors, CRC screening practices and/or improved treatments^1^. Several dietary components found in plant-derived foods, medicinal plants as well as their bioactive compounds have shown protective effects against a wide range of cancers, including colon cancer^2–4^. Therefore, it seems to be of relevance to identify new bioactive food or components with an anticancer potential to prevent and/or treat human cancers^5–7^.

Rosemary (*Rosmarinus officinalis* L.) is a bush of the Lamiaceae family that is mostly distributed in the Mediterranean area. In recent decades, experimental research has confirmed the pharmacological potential of rosemary and some of its primary compounds such as the diterpenes carnosic acid (CA) and carnosol (CAR), also expanding the range of its possible therapeutic applications. In fact, rosemary extracts have demonstrated chemoprotective effects against hepatotoxicity^8^ and gastric ulcerative lesions, and^9^ anticancer^10–13^, antimicrobial^14,15^, antioxidant^16^ and antidiabetic effects^17^, both *in vitro* and *in vivo*.

Recently, the antiproliferative effect of a terpenes-enriched rosemary extract (RE) obtained using supercritical fluid extraction has been demonstrated in colon cancer cell models^13^. A transcriptomic and metabolomic analysis in colon cancer cells indicated that RE treatment activated the expression of genes related to cell cycle progression and phase II antioxidant enzymes^18,19^. The bio-guided assay fractionation of the extract revealed that CA and CAR might be the main compounds responsible for such effects, but the higher activity of the complete extract compared to the fractions suggested the potential synergistic interaction between diterpenes and triterpenes. Nevertheless, the potential molecular mechanism of this antiproliferative effect and the pharmacological interactions between RE components are still unknown.

In the present report, the antiproliferative effect of RE has been deeply studied and the contribution of their different compounds to these effects thorough their pharmacological interaction has been characterized by synergy studies. The mechanism of the antiproliferative activity has been fully characterized by proliferation, migration, invasion and cell cycle assays in three different colon cancer cell lines. Moreover, the relationship between oxidative stress and cytotoxicity has been elucidated. Finally, we assessed the effect of RE on tumor progression *in vivo* in colon cancer mouse xenografts.

## Results

### Synergy studies

A previous study on the detailed composition of RE extract and the antiproliferative activity of their purified fractions in colon cancer cells revealed a putative pharmacological interaction between some of RE compounds^13^. This aspect was also pointed out by using a transcriptomic approach on some isolated compounds from RE such as CA and CAR in colon cancer cells^19^. Therefore, we decided to address this interaction by studying the putative synergistic effects between the major compounds in RE. We selected those compounds bearing the highest antiproliferative activities in previous studies, the diterpenes CA and CAR and the triterpenes betulinic acid (BA) and ursolic acid (UA) in single treatments or in pairwise combinations. First, individual IC_50_ values were determined for the antiproliferative effects of these four compounds compared to RE in HT-29 cells. The results show a dose-dependent antiproliferative effect (Supplementary Fig. 1) and that the triterpenes UA and BA exhibited higher antiproliferative effect than the diterpenes CA and CAR and all isolated compounds tested showed lower IC_50_ values than RE extract.

Furtherly, the synergistic interactions of these four compounds were profoundly scrutinized by using six pairwise combinations at different ratios. IC_50_ values for each combination were obtained and synergy was studied using three different methodologies: FICI value calculation, the graphic isobole method and the specialized software Compusyn. FICI values (Supplementary Table 1) showed additivity or an indifferent effect for all the combinations except for the BA-UA pair, which showed a clear antagonism behavior. Similar results were obtained using the isobole graphical method (Supplementary Figure 2), in which, no clear synergic behavior was observed for the selected ratios of the pairwise combinations of diterpenes. In contrast, antagonism was observed for the BA-UA combination. Only the Compusyn software results denoted a putative synergistic effect for different combinations between diterpenes and between di- and triterpenes, i.e. CA-CAR, CA-BA, CA-UA, CAR-UA, and CAR-BA (Supplementary Table 1). This synergistic effect was stronger in CAR-CA, CA-BA and CAR-BA combinations as shown in the polygonogram provided by the Compusyn software (Supplementary Figure 3). Again, BA-UA combination showed antagonism, as denoted in FICI calculations and isobole graphics. Taking all the synergy studies together, some pairwise combinations showed additive or synergic interactions depending on the approximation used what will be further discussed. However, the combination between the two triterpenes always brought antagonistic interaction no matter the method used.

However, no significant improvement in the antiproliferative activity was achieved when the complete extract was compared to the isolated compounds or their combinations. Therefore, for this reason, and due to its better availability, the subsequent studies were performed with the whole RE.

### RE inhibits tumor cell proliferation, colony formation and migration

To illustrate the antiproliferative effects of RE, basic cytotoxicity studies previously reported^13,19^ were further extended with complementary techniques focused to study cell proliferation, colony formation and migration in the three colon cancer cellular models.

**Figure 1:**
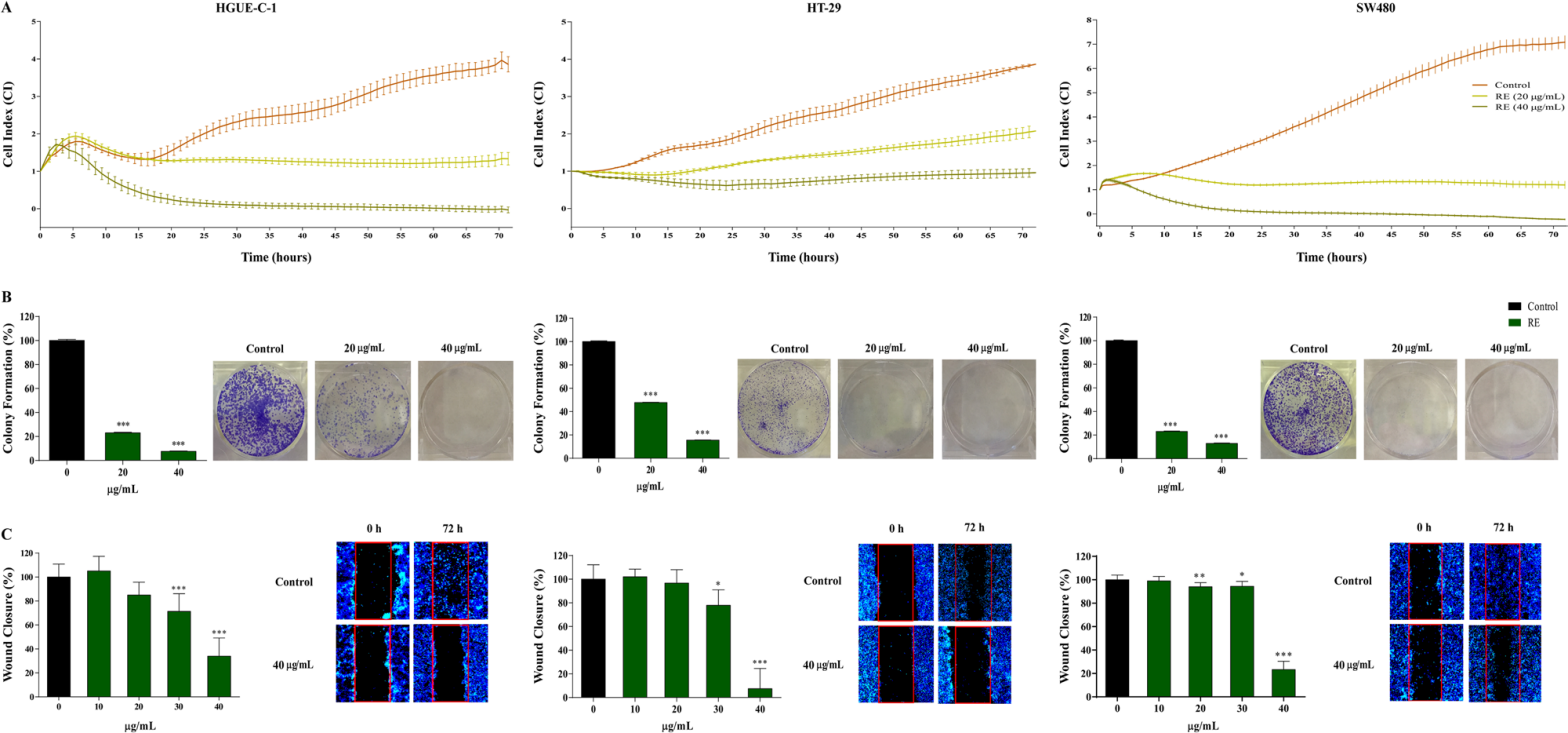
RE extract inhibits proliferation, migration and colony formation in human colon cancer cells. (**A**) Variation of cell index (CI) as a function of time for the three colon cancer cell lines, i.e. HGUE-C-1, HT-29 and SW480 in the absence (control) or in the presence of 20 µg/mL or 40 µg/mL of RE. Cell index was measured using the *xCELLigence* Real Time Cell Analysis System. (**B**) Inhibition of colony formation in the three colon cancer cell lines in the absence (control) or in the presence of 20 µg/mL or 40 µg/mL of RE. (**C**) Measurement of wound closure (%) by fluorescence imaging in wound healing migration assay for the three cell lines upon increasing concentrations of RE extract. Representative microscope images are also shown.

First, real-time cell proliferation was measured by RTCA as described in methods. Cells were treated with 20 and 40 μg/mL of RE for 72 h and cell growth curves were recorded by the *xCELLigence* System in real time (Fig. 1A). RE inhibited cell growth in a concentration dependent manner in all cell lines. HGUE-C-1 and SW480 cells demonstrated the highest sensitivity for RE, since a complete reduction of cell proliferation was observed already at 20 μg/mL, whereas some growth was observed for HT-29 cells at this concentration, in agreement to previous studies^13^. A cyclic growth pattern of the cell index parameter (valleys) was observed for the HGUE-C-1 and HT-29 cells, which correlated with the morphological changes of the cells during mitosis.

The clonogenic cell survival assay (colony formation assay) was employed to test whether RE was able to inhibit the ability of cancer cells to proliferate endlessly, so that retaining its reproductive capability to form a large colony or a clone (see methods section). Colorectal cancer cell lines were incubated with RE at different concentrations (20 or 40 µg/mL) for 7 days. RE suppressed the formation of colonies in the three colon cancer models in a dose-dependent manner (Fig. 1B). RE treatment at 20 and 40 µg/mL inhibited colony formation by 76.9% and 92.3%, respectively, in HGUE-C-1 cells when compared with the control; by 76.9% and 87.1%, respectively, in SW480 cells; and by 52.3% and 84.5% in HT-29 cells. Again, it was confirmed that HGUE-C-1 and SW480 cells were more sensitive to RE than HT-29 cells.

As migration capability is one of the central characteristics of metastatic cells^20^, the inhibitory effects of RE on the migration ability of HGUE-C-1, HT-29 and SW480 cells, was evaluated by using a wound healing assay (Fig. 1C). RE treatment at 30 and 40 µg/mL significantly inhibited cell migration by 28.8 and 66.1%, respectively, in HGUE-C-1 cells, when compared with the control. In HT-29 cells, cell migration was inhibited by 22.1% and 92.5%, and in SW480 cells, RE treatment inhibited cell migration 5.6% and 76.7%, at 30 and 40 µg/mL of RE, respectively.

### Studies on the mechanism of the antiproliferative effects of RE in colon cancer cells

#### Cell cycle and apoptosis

**Figure 2:**
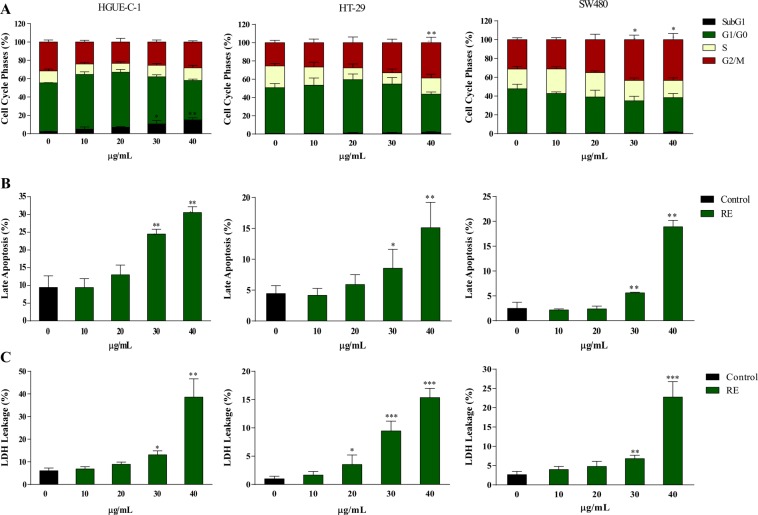
RE induces cell cycle arrest and cell death by necrosis in human colon cancer cells. (**A**) Cell cycle phases (%) analysis for the three colon cancer cell lines in the absence or in the presence of increasing concentrations of RE by Muse Cell Analyzer. (**B**) Measurement of late apoptosis in the three cell lines treated with the same concentrations of RE (%). (**C**) Plasma membrane integrity of the three cell lines treated equally and measured as LDH leakage (%).

To illustrate the putative mechanism of cell death in the different human colon cancer cell lines treated with RE, cell cycle and apoptosis analysis, mitochondrial viability, oxidative stress and necrosis measurements were performed. Figure 2A summarizes the results of cell cycle modulation after 24 hours of exposure to RE in the three different colon cancer cells lines. Significant decreases in G0/G1 phase with a concomitant accumulation of cells in the G2/M phase was observed in SW480 cells (at 30 and 40 µg/mL RE treatments, *p* < 0.05) and in HT-29 cells (and 40 µg/mL RE treatments, *p* < 0.01). In contrast, in HGUE-C-1 cells, RE decreased G0/G1 phase with accumulation of cells in SubG1 phase, showing an increase from 2.2% in control to 15.0% at highest concentration measured (*p* < 0.01).

Then, the effect of RE on apoptosis induction was specifically evaluated by detection of Annexin V-positive cells. HGUE-C-1, HT-29 and SW480 cell lines were stained with Annexin V/7-AAD and analyzed by Muse Cell Analyzer (Fig. 2B). RE treatment for 24 h substantially increased the fraction of Annexin V/7-AAD double-positive cells in a concentration-dependent manner, suggesting either that late apoptotic or necrotic death was occurring in all the cell lines. Although both late apoptotic and necrotic cells are Annexin V and 7-AAD positive, the absence of early apoptotic cells by cell cycle analysis (Supplementary Figure 4) suggests the presence of necrosis rather than apoptosis.

The expression of up to 43 apoptosis-related proteins was measured by using RayBio Human Apoptosis Array C1 (RayBiotech, Inc. USA,). In agreement to cell cycle analysis, only a few changes were observed in the three cell lines compared to the control (Supplementary Figure 5). HGUE-C-1 and SW480 cells showed a reduction in high temperature requirement A (HTRA) and survivin expression after RE treatment and an additional reduction in Bax protein was observed in SW480. No changes in these proteins were observed for HT-29 upon RE treatment. Taken together, all these results suggest that apoptosis was no the main death mechanism.

#### Necrosis studies

The release of the intracellular enzyme LDH due to permeabilization of the plasma membrane is a hallmark of necrosis^21^. LDH leakage assay is a simple, reliable and fast cytotoxicity assay based on the measurement of LDH activity in the extracellular medium^22^. RE treatment of cancer cells induced a dose-dependent release of LDH compared to the control (Fig. 2C). After 24 h of RE treatment at 40 µg/mL, HGUE-C-1 and SW480 cells exhibited the highest increase (32.6% and 20.0% of control, respectively), whereas an increase of 14.3% was observed in HT-29 cells.

The morphological observation of RE treated cells by microscopy (data not shown) also suggested a necrosis rather than apoptosis mechanism. However, as necroptosis, a mixed mechanism between necrosis and apoptosis and a caspase-independent form of regulated cell death^23,24^, shares some morphologic features with necrosis, this option was also evaluated. To study this hypothesis, the RIP1 inhibitor necrostatin-1 (Nec-1)^25^ was used. Pretreatment of cells with Nec-1 before RE exposure did not suppress cell death (Supplementary Figure 6A). Since autophagy has been involved as a regulator for both apoptotic and non-apoptotic cell death, the potential presence of autophagy was also evaluated. Cells were pretreated with chloroquine (CQ), a well-known autophagy inhibitor^26^, but no changes were detected compared to cells treated with RE in the absence of CQ (Supplementary Figure 6B). These findings confirmed that RE inhibits colon cancer cell proliferation most probably by inducing necrosis as the major death mechanism.

#### Intracellular ROS generation and mitochondrial membrane potential measurements

**Figure 3:**
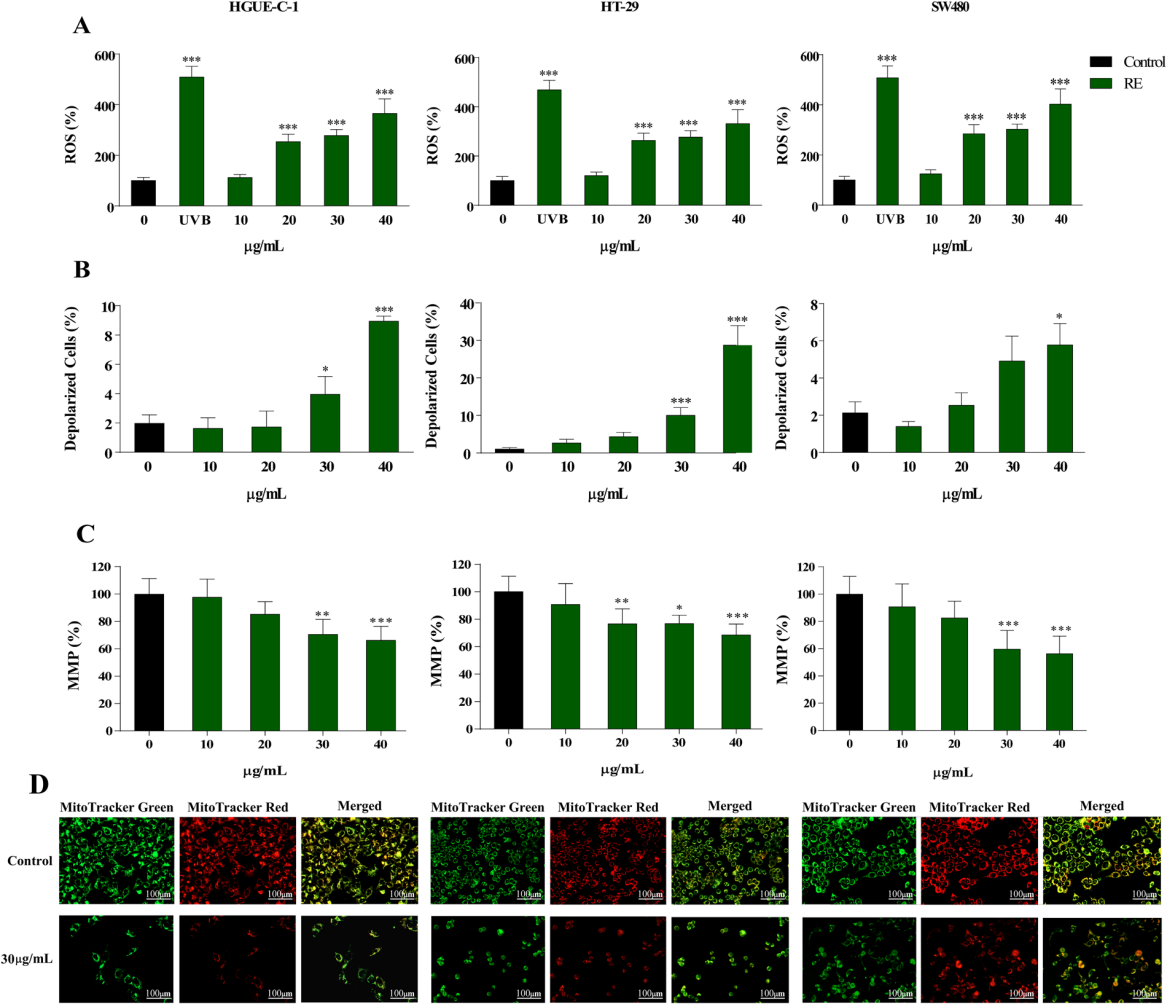
RE produces ROS generation and mitochondrial membrane depolarization in human colon cancer cells. (**A**) Measurement of intracellular ROS levels (%) of the three colon cancer cell lines after UV radiation in the absence or in the presence of 10, 20, 30 or 40 µg/mL RE, using H_2_DCFDA dye. (**B**) Quantitation of depolarized cells (%) in the three cell lines treated with the same concentrations of RE using Muse Cell Analyzer and (**C**) MMP measurement (%) by using MitroTracker Green FM and MitoTracker Red CMXRos fluorescent dyes. (**D**) Representative fluorescence images of HGUE-C-1, HT-29 and SW480 cells, stained with MitoTracker Green and MitoTracker Red are shown.

Rosemary compounds have exhibited the capacity to regulate oxidative stress in different *in vitro* and cellular systems^16,27^. In addition, both apoptosis and necrosis have been shown to be triggered by ROS^28^. Therefore, we determined whether the treatment of colon cancer cells with RE could modulate intracellular ROS. ROS accumulation was evaluated in the three colorectal cancer cells using the non-polar cell-permeable probe H_2_DCFDA. As shown in Fig. 3A, the treatment with RE induced an increase in fluorescence intensity for all the colorectal cancer cell lines, indicating an increase of ROS generation in a concentration-dependent manner.

Mitochondrial membrane integrity and mitochondrial viability are impaired in necrotic cell death^29^. In addition, mitochondrial function is closely related to ROS production and control. To deepen into the effects of RE treatment on mitochondrial viability, the mitochondrial membrane potential variation was determined with the Muse Cell Analyzer, using the Muse MitoPotential kit. Figure 3B shows a dose-response enhancement in the number of cells bearing depolarized mitochondrial membranes with the increase of RE concentration that suggests a mitochondrial depolarization, which could be linked with the activation of necrosis in colorectal cancer cells. Alternatively, MitoTracker Red CMXRos and MitoTracker Green fluorescent probes were also used to examine whether mitochondrial membrane potential was injured by RE treatment in colorectal cancer cells. A decrease of the ratio between red and green fluorescence indicated a loss of mitochondrial membrane potential, as occurred when all colon cancer cells were treated with RE in a dose-dependent manner, especially in SW480 cells, (Fig. 3C,D), and corroborating the results obtained in Fig. 3B.

#### Nrf2 modulation by RE treatment in colorectal cancer cells

**Figure 4:**
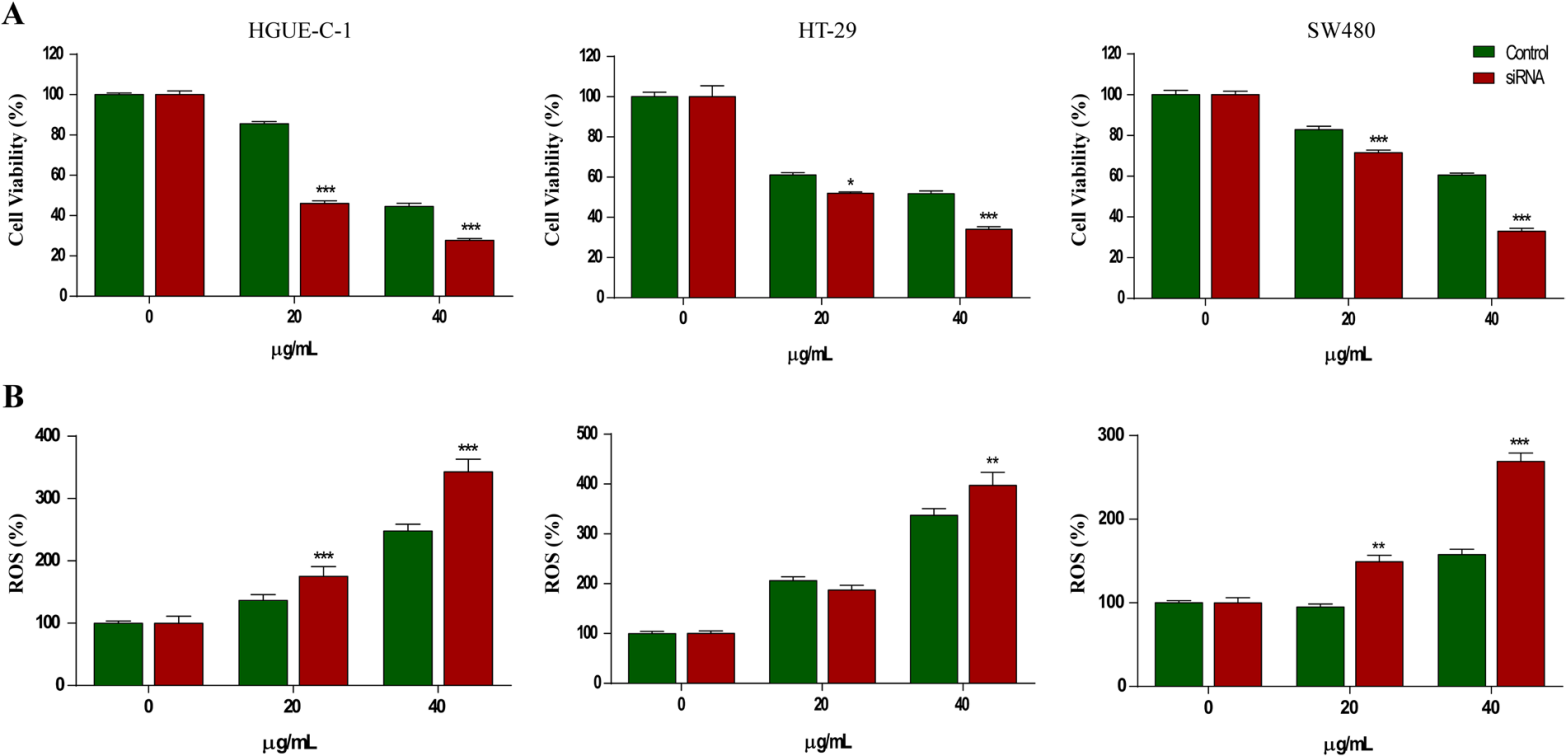
Nrf2 transcription factor silencing increases RE-induced cell death in colon cancer cells. (**A**) Measurement of cell viability (%) of the three colon cancer cell lines, i.e. HGUE-C-1, HT-29 and SW480 in the absence (control) or in the presence of 20 µg/mL or 40 µg/mL of RE, in the absence or in the presence of an Nrf2 specific siRNA. (**B**) Measurement of intracellular ROS levels (%) in the three cell lines treated in the same conditions as above using H_2_DCFDA dye.

It is known that intracellular ROS generation affects the function of multiple redox-sensitive transcription factors and leads to the up-regulation of antioxidant genes. Cellular antioxidant defense mechanisms include the ROS scavenger molecules, phase II detoxification enzymes, and other detoxifying proteins^30,31^. The transcription factor Nrf2 is a key regulator of numerous detoxifying and antioxidants genes and is activated in response to oxidative and electrophilic stress and its relationship with RE mechanism was studied by silencing it using a specific siRNA (see methods section and Supplementary Figure 7). As shown in Fig. 4, Nrf2 gene silencing in HGUE-C-1, HT-29 and SW480 cells did not modify neither viability or ROS generation in the absence of RE. Nevertheless, when cells were treated with RE, Nrf2 silencing induced a significant decrease in cell viability (Fig. 4A) concomitantly with an increase in ROS production (Fig. 4B), both in a dose-dependent way.

### *In vivo* experiments

#### Oral Acute Toxicity study

The acute oral toxicity study or RE was performed according to the Organization for Economic Cooperation and Development (OECD) guideline 420, which specifies a limit test dose of 2000 mg/kg. After 17 days, no lethal effects or signs of toxicity were observed in any the groups of rats treated with different doses (300 and 2000 mg/kg) of the RE. No significant differences were observed in the body weight of animals treated with RE compared to the control (Supplementary Figure 8), being this parameter one of the first critical signs of toxicity and is often the most sensible indicator of an adverse effect^32^. In addition, there were no significant behavioral changes, such as apathy, hyperactivity or morbidity in any of the animals when compared to the control. Normal increase in body weight was recorded for all animals with no abnormalities at necropsy on day 17^th^. Non-altered growth or abnormal changes were detected during the macroscopic analysis of the rat organs. For the histopathology analysis, the organs including spleen, heart, liver, lungs and kidney were examined. No pathological changes were noticed in neither the RE-treated groups nor the control group at the end of experiment (Supplementary Figure 9). Therefore, according OCDE 420 guide, the LD_50_ of extract may be greater than 2000 mg/kg.

#### *In vivo* effects of RE on tumorigenicity of HT-29 cells in athymic mouse model

**Figure 5:**
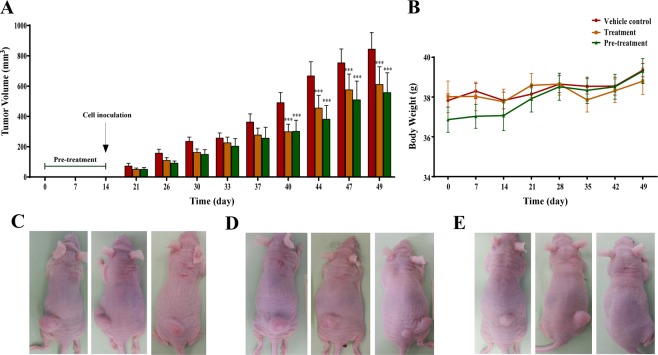
RE oral administration decreases tumor volume in athymic nude mice. (**A**) Tumor volumes (mm^3^) in the absence (vehicle control), pre-treatment (two weeks oral administration of RE before cell inoculation) or treatment (administration of RE after cell inoculation) groups at doses of 200 mg/kg of RE (oral administration) in a xenograft model of colon cancer cells using athymic nude mice. Animals were monitored twice a week during 35 days. (**B**) The body weight (g) of the animals was recorded during the assay for the three groups. Representative images of tumor formation in (**C**) vehicle-control, (**D**) treatment and (**E**) pre-treatment groups.

Having identified significant *in vitro* antiproliferative and necrotic activity and the putative mechanism of RE antiproliferative effects, the next step was to determinate whether these results could be translated to an *in vivo* model. For this purpose, HT-29 cells were implanted in athymic nude mice and two different oral treatments with RE were assayed: a) 2 weeks pretreatment + cells inoculation + treatment; b) cells inoculation + treatment. To assess overall general health and well-being of animals during treatment, body weights were recorded once a week. RE-treatment did not cause any loss in body weight (data not shown) or change in food intake and there were no apparent signs of toxicity in animals according the welfare list punctuation approved by the ethical committee. The average volume of tumors in control mice increased as a function of time and reached an end point of 1,000 mm^3^ in 35 days post-inoculation. As shown in Fig. 5, both treatments significantly reduced the measurements of tumor volume. RE treatment for 7 weeks (2 weeks pre-treatment + 5 weeks treatment) significantly reduced tumor volume by 34.1% (*p* < 0.001), whereas RE treatment after cells inoculation reduced tumor volume 27.5% (*p* < 0.001).

## Discussion

This work was intended to deepen into the mechanism of our previous research that showed the antiproliferative activity of a well characterized rosemary extract in correlation to its effects on the gene expression of colon cancer cells using transcriptomic and metabolomics analysis^13,18,19^. Our previous research characterized in detail by HPLC-ESI-QTOF-MS the composition of the RE utilized in the present work, showing that diterpenes (CA and CAR) and triterpenes (UA and BA) were the most abundant compounds^13^. This study also revealed that the antiproliferative capacity of the RE was stronger than that of their isolated fractions so cell studies on the putative synergistic effects of single compounds present in the extract should be undertaken. Therefore, in this work, potential synergistic effects among four of the most abundant compounds in the extract, two diterpenes, CA and CAR, and two triterpenes (UA and BA) were suggested. When the four terpenoids were individually considered, significant antiproliferative activity was observed for each one, in correlation with previously reported data (reviewed in^13^). The triterpenes antiproliferative activity was higher than that of diterpenes and this, in turn, a little higher than that of the whole RE. When these four compounds were studied in pairwise combinations, interesting pharmacological interactions between them were discovered although the different methods used brought some differences.

As shown by FICI and Compusyn methods, most of the pairwise combinations between di- and triterpenes showed additivity or mild synergy, which could explain the higher activity of RE when compared with individual and combined compounds. In contrast, the triterpene combination UA-BA always brought antagonism no matter the method used, indicating a putative competitive mechanism between them. Nevertheless, this antagonism did not seem to be strong enough to counterbalance the additive or synergic interaction between diterpenes and between di and triterpenes in the whole extract. Given that the isobole method is a semiquantitative and graphical approach, the results obtained through this method may be questionable^33^. In contrast, FICI calculation and Compusyn software gave more consistent results between them, and their differences could be attributed to the different mathematical approach used. The polygonogram obtained by the Compusyn software (Supplementary Figure 3) summarizes the types of interactions between the main terpenes present in the extract, i.e. antagonism between the triterpenes BA-UA, a strong synergetic behavior between the diterpenes CAR and CA, but also synergism between diterpenes and triterpenes, i.e. CAR-UA, CAR-BA, CA-UA and CA-BA.

Therefore, these interactions may account for the strong antiproliferative capacity of whole RE. In this regard, a synergetic behavior between compounds in natural extracts has been also described not only for anticancer activity^34,35^ but also for antimicrobial^36^ or antilipidemic^37^ activities.

In an attempt to illustrate the mechanism of the *in vitro* antiproliferative capacity of RE, a set of assays were utilized to determine the capacity of RE to modulate several signatures of tumorigenesis such as proliferation, migration and invasion. Previous studies using the same RE showed a dose-response decrease of colon cancer cell viability^13^. RTCA experiments confirmed that the inhibition of cell proliferation was one of the effects exerted by RE, which could be complemented by a cytotoxic effect. This antiproliferative effects of RE were also confirmed in the three colon cancer cell models in a dose response manner, regardless of the different pheno and genotype of the cell line.

We next investigated whether RE is able to inhibit *in vitro* several signatures of cancer metastasis such as migration, invasion and clonogenic survival. In metastasis, cancer cells with epithelial phenotype migrate away from primary tumor crossing the basement membrane, a network of extracellular matrix, and later migrate through the stroma reaching blood or lymph vessels, where they can be carried to other organs^38^. Once in other tissues, their invasiveness will depend on their clonogenic survival. RE severely reduced both, the cell migration in a wound-healing assay and the clonogenic survival of the three colon cancer cells in a dose-response manner, indicating that RE would not be able only to reduce tumor growth and proliferation but also their invasion and migration capabilities, which indicates a lower metastatic potential as the main hallmark of cancer.

Our cell cycle results indicate alterations of the cell cycle by RE such as late apoptosis and a decrease in G0/G1 phase with a concomitant accumulation of cells in the G2/M, especially in HT-29 and SW480 cell lines, in agreement to previously reported^18,19,34,39^. The analysis of a panel of apoptosis-related proteins revealed that RE did not substantially change the expression of these proteins (Supplementary Figure 5). Only mild but statistically significant decrease was observed for two proapoptotic proteins (Bax and HTRA), although, with no biological relevance. A more relevant decrease was obtained for survivin in HGUE-C-1 and SW480 cells. Survivin plays an important role in cancer development, and it has been involved in the resistance of tumor cells to both radiotherapy and chemotherapy^40^. A recent meta-analysis showed that the upregulation of survivin is associated with poor prognosis in patients with colorectal cancer^41^. Nevertheless, these alterations in cell cycle and apoptosis proteins do not seem to justify the strong antiproliferative capacity of RE.

Further experiments using LDH leakage confirmed that necrosis was the main death mechanism (Fig. 2C), which was confirmed by morphological observation. This necrosis mechanism was also concomitant to a dose-response increase of intracellular ROS, the loss of mitochondrial potential and the activation of the Nrf2 pathway, as confirmed by Nrf2 gene silencing experiments (Fig. 4). These results confirm previous metabolomic studies that show increased expression of antioxidant enzymes and activation of Nrf2 pathway in HT-29 cells treated with CA^19^. These results also may offer new opportunities for alternative targeted antitumor therapies based on the combination of rosemary compounds and small Nrf2 inhibitors for certain types of cancers^42^.

Once the antiproliferative capacity as well as the inhibition of migration and invasion of colon cancer cells by RE was demonstrated *in vitro*, we examined its inhibitory role on tumor growth in a xenograft model of HT-29 colon cancer cells. The capacity of RE (200 mg/kg three times a week, p.o.) to reduce tumor size was demonstrated in a four weeks assay. Further, oral pretreatment for two weeks before cell inoculation followed by the treatment seemed to be more effective than just the treatment itself. These results confirmed those reported with other rosemary extracts and/or compounds using xenografts of SW620 or HCT116 colon cancer cells in athymic nude mice^12^. Although preliminary bioavailability studies point out that diterpenes, and especially CA, show higher absorption and permeability than other rosemary compounds^43,44^, further studies must be done to identify the potential metabolites responsible for such anticancer effects. As part of the preclinical approach, the histopathological and macroscopic analysis confirmed the absence of toxicity in an oral acute toxicity assay according OCDE 420 guidance. According to the Globally Harmonized System (GHS) of Classification and Labelling of Chemicals, RE was rated as non-classified substance at a dose of 2000 mg/kg^45^, as reported for other rosemary extracts^46^.

Previously reported studies in colon cancer cells postulate that rosemary compounds exert antiproliferative effects by inhibiting proliferative and survival signaling pathways such as PI3K/Akt, as well as modulating Nrf2 transcription factor pathway^18,47^. Apoptosis and cell cycle arrest has also been proposed as part of the antiproliferative mechanism of RE^18,48,49^. Nevertheless, our study suggest that necrosis rather than apoptosis is the mechanism to account for colon cancer cell death. In agreement to our results, a recent proteomic analysis in colon cancer cells also revealed that RE altered proteins implicated in the activation of Nrf2 transcription factor and the unfolded protein response (UPR)^48^.

In this work, we have corroborated the prooxidant effects of rosemary compounds, recently reported by our group^47^, as mediator of their antiproliferative effects. According to our results and those previously reported, we postulate that ROS generated by RE in colon cancer cells may be responsible for an exacerbated UPR response and endoplasmic reticulum stress (ERS) leading to the activation of Nfr2, apoptosis and autophagy as defense mechanisms. Under this scenario, autophagy may be unable to counter excessive redox imbalance and cellular stress, and cell homeostasis evolves into necrotic cell death. Targeting colon cancer cells by increasing intracellular ROS and decreasing cell survival response may increase the therapeutic potential of RE compounds as in combination to chemotherapeutic drugs.

In conclusion, we reveal that RE compounds show the *in vitro* capability to inhibit cellular proliferation, migration and invasiveness of colon cancer cells. The treatment of cancer cells with RE leads to a strong increase of intracellular ROS that results in necrosis cell death. According to our results, Nrf2 transcription factor pathway seems to be involved in cell survival upon RE treatment. These *in vitro* results were in line with a reduction of tumor growth by RE in a xenograft model of colon cancer cells in athymic nude mice. Whether similar antiproliferative mechanism takes place *in vivo* or not, requires preclinical studies to correlate the presence of rosemary metabolites with metabolic biomarkers.

## Materials and Methods

### Chemicals and cell culture

All chemicals, reagents and the rosemary extract obtained by supercritical fluid extraction are described in detail in the Supplementary information. Cells were grown in DMEM supplemented with 5% heat-inactivated FBS, 2 mM L-glutamine, penicillin-streptomycin (0.1 mg/mL penicillin and 100 U/mL streptomycin) at 37 °C in a humidified atmosphere with 5% CO_2_. Colon adenocarcinoma HT-29 and SW480 cells were obtained from the IMIM (Institut Municipal d’Investigació Médica, Barcelona, Spain) and ATCC (American Type Culture Collection, LGC Promochem, UK), respectively, and HGUE-C-1 was an established cell line derived from a primary colon cancer cell line of a single primary human colon carcinoma at the Hospital General Universitario de Elche (Alicante, Spain), as described^13^. The cells were trypsinized every three days and they were seeded in 96- or 6-well plates depending on the assay^13^.

### Synergy studies

The concentration that inhibited 50% of the cell growth (IC_50_ value) of every single compound and those of their pairwise combinations were estimated by 3-(4,5-dimethylthiazol-2-yl)-2,5-diphenyltetrazolium bromide (MTT) assay as previously described^13^ (see Supplementary material for detailed methodology). The results of synergy studies were interpreted by using three different methods: fractionated inhibitory concentration index (FICI), Isobolographic method and Compusyn software^50^. Further information about these methods to determine synergy is provided in the Supplementary information.

### Cell cycle, apoptosis and mitochondrial membrane potential (MMP) analysis

Human colorectal cancer cell lines (15 × 10^4^ cells/well) were seeded into 6-well plates. After 24 h of culture, cells were treated with different concentrations of RE (10–40 µg/mL) for 24 h. Afterwards, the treatment, cell cycle phase pattern, apoptosis and mitochondrial membrane potential were determined by using different kits for Muse Cell Analyzer (Merck-Millipore, Darmstadt, Germany) according to the manufacturer´s instructions. Additional information on these procedures is provided in Supplementary material. MMP was also evaluated by a different method using MitoTracker Red CMXRos and MitoTracker Green fluorescent probes, which is also detailed in Supplementary information.

### Measurement of plasma membrane integrity

A cytotoxicity detection kit based on the extracellular detection of lactate dehydrogenase (LDH) was used to monitor cell integrity. Human colorectal cancer cell lines (5 × 10^3^ cells/well) were seeded into 96-well plates. Cells were treated for 24 with RE (10–40 µg/mL), centrifuged, and 100 μL of the cell culture supernatant transferred to a clean 96-well plate. The LDH detection reagent was added and incubated for 15 min in the dark, and absorbance read at 492 nm (reference filter 600 nm). LDH leakage (% cytotoxicity) was calculated as follows:

$${\rm{Cytotoxicity}}( \% )=\frac{\exp .\,{\rm{value}}-{\rm{low}}\,{\rm{control}}}{{\rm{high}}\,{\rm{control}}-{\rm{low}}\,{\rm{control}}}\,\times \,100$$

where *low control* represents LDH activity released from the untreated normal cells (spontaneous LDH release) and *high control* represents maximum releasable LDH activity in the cells (maximum LDH release).

### Evaluation of the intracellular reactive oxygen species (ROS) generation

Intracellular ROS levels were measured using 2′,7′-dichlorofluorescein diacetate (H_2_DCFDA) probe (Molecular probes, Life Technologies Co., Europe). Human colorectal cancer cell lines (5 × 10^3^ cells/well) were seeded into 96-well black plates. Cells were treated for 24 h with RE (10–40 µg/mL) and probed with 10 µg/mL of H_2_DCFDA. Fluorescence was detected by excitation at 485 nm and emission at 535 nm using a microplate reader (POLARstar Omega, BMG LabTech GmbH, Offenburg, Germany).

### RNA interference assay

Cells were transfected with siRNA specific for Nrf2 by using Lipofectamine (Invitrogen, Europe) according to the manufacturer’s instructions. The silencing ability of all siRNAs was determined by Dot Blot analysis (see Supplementary information for details). At 24 h post-transfection, cells were treated with the RE (20 or 40 µg/mL). After treatment cell viability, ROS level and protein expression were studied.

### Wound healing assay

The ability of cells to migrate was assayed by wound healing assay. Human colorectal cancer cell lines were seeded in a 96-well plate and incubated at 37 °C. When confluent, cells were scratched with a 10-μL pipette tip, followed by washing with PBS. The cells were then treated with RE in complete medium for 72 h. Cells were stained with Hoechst 33342 reagent, and the population of cells that migrated into the scratched area was quantified by Cytation 3 Cell Imaging Multi-Mode Reader (BioTek, Germany). Quantitation of the migrated cells was measured using Image J software. Wound healing rate was assessed by calculating the wound area fraction between wound width at 0 and 72 h.

### Real-Time Cell Analysis (RTCA) proliferation assay

Cell proliferation was studied using RTCA DP instrument *xCELLigence* system (Roche Diagnostics GmbH, Germany), which was placed in a humidified incubator maintained at 5% CO_2_ at 37 °C. Thereafter, 7500–40000 cells (depending on cell line) were added to each well of E-plates (Roche Diagnostics GmbH, Germany) and were incubated at room temperature to allow the cells to settle. The plates were then placed in the *xCELLigence* system. After 24 h, cells were treated with RE (20 or 40 µg/mL) for 72 h. Cell growth and proliferation were monitored through the cell index (CI) values, provided by the instrument, and data analysis was performed with the supplied RTCA software.

### Colony formation assay

Human colorectal cancer cell lines cells (5 × 10^3^ cells/well) were seeded into 6-well plates. After being attached to the plate, the cells were treated at 20 or 40 µg/mL of RE for 24 h and then cultured with fresh medium for 7 days. After that, medium was removed, and cells washed twice with PBS. The colonies were fixed with 95% ethanol for 10 min, dried and stained with 0.1% crystal violet solution for 10 min, and the plate was washed three times with water. Colonies on the plate were counted under a microscope, and each colony consisted of more than 50 cells.

### *In vivo* experiments

An acute oral toxicity test was performed as described in Organization for Economic Cooperation and Development (OECD) 420 guideline using Fixed Dose Procedure to minimize the use of animals (see details in the Supplementary information).

*In vivo* antiproliferative activity of RE was determined by using male nude mice in which, xenografts of human colon cancer cells (HT-29) were established subcutaneously. Animals were randomly divided into three groups (n = 10–12). In the first group, animals were treated orally with RE for two weeks before cell inoculation. In the second group, RE was administered to animals after cell inoculation, and in the last group (control), animals were treated only with the vehicle. Refer to additional information in Supplementary information.

The *in vivo* experimental protocols ware approved by Ethics Committee of the Miguel Hernández University (references IBM-VMM-001-11 and UMH-IBM-VMM-02-14), according to the Spanish and European regulation and animal welfare guides.

### Statistical Analysis

The data were expressed as the mean ± standard deviation (SD) of 4–10 determinations, depending on the assay. One-way analysis of variance (ANOVA) and statistical comparisons of the different treatments were performed using Tukey’s test in GraphPad Prism version 5.0 (GraphPad Software)^27^. Data are expressed as mean ± SD. *(*p* < 0.05), **(*p* < 0.01) or ***(*p* < 0.001) indicate statistically significant differences compared to control unless otherwise stated.
